# Field-based high-throughput phenotyping enhances phenomic and genomic predictions for grain yield and plant height across years in maize

**DOI:** 10.1093/g3journal/jkae092

**Published:** 2024-05-22

**Authors:** Alper Adak, Aaron J DeSalvio, Mustafa A Arik, Seth C Murray

**Affiliations:** Department of Soil and Crop Sciences, Texas A&M University, College Station, TX 77843-2474, USA; Interdisciplinary Graduate Program in Genetics and Genomics (Department of Biochemistry and Biophysics), Texas A&M University, College Station, TX 77843-2128, USA; Department of Soil and Crop Sciences, Texas A&M University, College Station, TX 77843-2474, USA; Department of Soil and Crop Sciences, Texas A&M University, College Station, TX 77843-2474, USA

**Keywords:** phenomic prediction, genomic prediction, multikernel prediction, field-based high-throughput phenotyping, UAV, functional principal component analysis, maize breeding, grain yield, plant height

## Abstract

Field-based phenomic prediction employs novel features, like vegetation indices (VIs) from drone images, to predict key agronomic traits in maize, despite challenges in matching biomarker measurement time points across years or environments. This study utilized functional principal component analysis (FPCA) to summarize the variation of temporal VIs, uniquely allowing the integration of this data into phenomic prediction models tested across multiple years (2018–2021) and environments. The models, which included 1 genomic, 2 phenomic, 2 multikernel, and 1 multitrait type, were evaluated in 4 prediction scenarios (CV2, CV1, CV0, and CV00), relevant for plant breeding programs, assessing both tested and untested genotypes in observed and unobserved environments. Two hybrid populations (415 and 220 hybrids) demonstrated the visible atmospherically resistant index’s strong temporal correlation with grain yield (up to 0.59) and plant height. The first 2 FPCAs explained 59.3 ± 13.9% and 74.2 ± 9.0% of the temporal variation of temporal data of VIs, respectively, facilitating predictions where flight times varied. Phenomic data, particularly when combined with genomic data, often were comparable to or numerically exceeded the base genomic model in prediction accuracy, particularly for grain yield in untested hybrids, although no significant differences in these models’ performance were consistently observed. Overall, this approach underscores the effectiveness of FPCA and combined models in enhancing the prediction of grain yield and plant height across environments and diverse agricultural settings.

## Introduction

In recent decades, the application of field-based high-throughput phenotyping (FHTP) has seen a significant rise across various fields and staple crops (Herr *et al*. 2023). This emerging approach has allowed scientists to collect extensive data efficiently to better understand plant biology in relation to plant growth dynamics. FHTP data such as LiDAR and image-based orthomosaics are desired due to their ability to capture essential plant characteristics such as height, shape, canopy structure, and crop health. Such data have been instrumental in (1) examining lodging in relation to yield and plant height (Chu *et al*. 2017; Cen *et al*. 2019; Singh *et al*. 2019; Sun *et al.* 2019; Zhao *et al*. 2020; Zhou *et al*. 2020; Tirado *et al*. 2021; Sun, Gu, *et al*. 2022; Liu *et al*. 2023), (2) assessing plant growth in response to different managements and environmental stimuli using temporal plant height data (Anderson *et al*. 2019; Tirado *et al*. 2020; Adak, Anderson, *et al*. 2023), (3) identifying genes that regulate the temporal growth of plant height, canopy, nitrogen response, early growth rate, and vegetation indices (VIs) at multiple time points during growth (Pauli *et al*. 2016; Xavier *et al*. 2017; Condorelli *et al*. 2018; Wang *et al*. 2019; Anderson *et al*. 2020; Miao *et al*. 2020; Ogawa *et al*. 2020; Parker *et al*. 2020; Adak *et al*. 2021; Adak, Murray, Anderson, *et al*. 2021; Wang, Li, *et al*. 2021; Rodene *et al*. 2022; Adak *et al*. 2023; Ye *et al*. 2023), (4) estimating nitrogen traits in relation to photosynthesis, biomass, and crop yield (Buchaillot *et al*. 2019; Corti *et al*. 2019; Colorado *et al*. 2020), (5) dissecting time-dependent associations belonging to specific time points of growth between temporal phenotype, genomic, and environmental data (Wang, Li, *et al*. 2021; Sun, Lu, *et al*. 2022; Adak *et al*. 2023), and (6) evaluating the genetic basis and temporal impact of biotic and abiotic stresses such as lodging, drought, and heat stress (Singh *et al*. 2016; Jiang *et al*. 2021; Chowhan and Chakraborty 2022; Li *et al*. 2022; Adak *et al*. 2023).

As demonstrated by these research findings, the wide capabilities of FHTP paved the way for a paradigm shift to phenomic prediction. FHTP data enable plant growth to be observed in a quantitative manner as each genotype responds to the environment, using repetitive drone (unoccupied aerial vehicles [UAVs]) flights equipped with high tech sensors (unoccupied aerial systems [UASs]), providing a main source of biomarkers in phenomic prediction of complex traits (Herr *et al*. 2023; Yoosefzadeh-Najafabadi *et al*. 2023). Phenomic data have been effectively integrated into prediction models, serving as secondary traits in genomic prediction, primary predictor data, or combined with genomic data to forecast complex traits in crops like maize, wheat, and rye (Rutkoski *et al*. 2016; Aguate *et al*. 2017; Crain *et al*. 2018; Krause *et al*. 2019; Wu *et al*. 2019; Sun, Poland, *et al*. 2019; Galán *et al*. 2020; Krause *et al*. 2020; Adak, Murray, Božinović, *et al*. 2021; Adak *et al*. 2022; Adak *et al*. 2023). For instance, studies have demonstrated that incorporating drone-derived features as secondary traits in genomic prediction models enhances the accuracy of grain yield (GY) predictions compared to univariate genomic prediction methods (Crain *et al*. 2018; Sun, Poland, *et al*. 2019; Krause *et al*. 2020). Additionally, research has shown that utilizing temporal information from drone-derived features across various growth stages enables accurate GY predictions, performing at a level comparable to genomic prediction in maize (Adak, Murray, Božinović, *et al*. 2021; Adak *et al*. 2022; Winn *et al*. 2023). Moreover, the integration of multikernel models incorporating both genomic and phenomic information has shown superior to univariate genomic prediction (Crain *et al*. 2018; Galán *et al*. 2020; Adak *et al*. 2023). However, a deficit exists in the realm of phenomic predictions conducted across years and diverse environments, each characterized by disparate and incongruent flight schedules. The predictor biomarkers do not necessarily align by flight date or stage of crop development. This deficit is a significant gap in deploying phenomic predictions, where predictive modeling with temporal phenomic data in varied contexts remains underexplored. This scarcity of comprehensive studies hinders understanding of how phenomic traits evolve over time, in response to different environmental factors, and is confounded by varying flight timing. Addressing this research gap could lead to more nuanced insights into the dynamic interplay between phenomic feature biomarkers and environmental conditions, ultimately enhancing the precision and applicability of predictive models in predictive plant breeding or fundamental plant biology.

Temporal phenotyping allows for the observation and measurement of traits over time, rather than at a single time point. This is crucial for complex traits like yield or plant height, which are influenced by the cumulative effects of genetic and environmental factors throughout the growth period. By analyzing these traits over their entire developmental timeline, researchers can gain a more accurate and comprehensive understanding of the genetic factors that contribute to final yield or agronomic traits, such as terminal plant height. Complex traits are often the result of interactions between genes and environmental factors. Temporal phenotyping and phenomics facilitate the study of these interactions over time, helping to identify which genetic factors are influential under specific environmental conditions. This understanding is critical for predicting yield and plant height in varying climatic and soil conditions. Temporal phenotyping helps in understanding phenotypic plasticity, which is the ability of an organism with a given genotype to change its phenotype in response to environmental conditions. This is particularly important for crops, as it can inform breeding programs about which genotypes are most adaptable to changing environmental conditions, a key factor in yield stability. Genomic selection, a method used in animal and plant breeding, relies heavily on the prediction of complex traits. Temporal phenotyping provides a wealth of phenotypic data over time, which can be integrated with genomic data to improve the accuracy of these predictions. This is especially useful for traits that are expressed or become measurable at different stages of development. Some traits contributing to yield may only be expressed or observable at certain stages of development. Temporal phenotyping allows for the identification and quantification of these stage-specific traits, which can then be targeted in genetic and genomic studies for crop improvement or biological discovery. Incorporating temporal phenotypic data into predictive models for complex traits like yield can significantly enhance their accuracy. These models become better equipped to account for the dynamic nature of trait development and environmental interactions, leading to more reliable predictions.

Functional principal component analysis (FPCA) is a statistical technique used to analyze functional data, where the data points are curves or functions in time rather than traditional scalar or vector data (Yao *et al*. 2005). It extends the concept of traditional principal component analysis (PCA) to functional data by extracting principal components from a set of curves or functions in time. In remote sensing, FPCA can analyze spectral data collected from different wavelengths over time. It can identify the most significant patterns or variations within this multidimensional spectral data. This enables the identification of key features or behaviors that represent changes in vegetation health or environmental conditions. It separates the signal (important variation) from noise (less relevant variations or measurement errors) within functional time series data, providing clearer understanding of underlying patterns. Overall, the use of FPCA in remote sensing offers a powerful approach to analyze and interpret disparate time-varying data captured by sensors, enabling researchers to extract meaningful information, reduce noise, and improve the understanding and prediction of changes in experimental area over time (Ramsay and Silverman 2002).

The main objective of this study was 3-fold: first, to employ FPCA on temporal phenomic features extracted from drone images. This analysis was applied to conduct phenomic predictions across multiple environments and years, specifically for predicting plant height and GY in maize. These predictions were evaluated under varied prediction scenarios, involving both tested genotypes (dependent variables utilized in training models) and untested genotypes (dependent variables not included in training models) in both tested and untested environments. Second, the study aimed to develop prediction models using multikernel approaches incorporating both genomic and temporal phenomic data. It sought to explore multitrait genomic prediction methods by incorporating a summarized FPCA phenomic feature as a secondary trait in prediction. Additionally, temporal correlations between different VIs and agronomic traits (yield and plant height) were examined.

## Materials and methods

### Population structure and growing condition

Two different maize hybrid populations from the genomes to field project (G2F; https://www.genomes2fields.org/) were used in this study, and both populations were grown in College Station, Texas. The first population (Pop1) was developed by crossing doubled-haploid-derived inbred lines with LH195 a stiff stalk tester (Foleyand Holden's Foundation Seeds, Inc. 1991). The doubled-haploid inbreds, including PHW65 (common parent), PHN11, Mo44, and MoG, were used. Pop1 consisted of 415 subset hybrids planted on 2018 March 6 and 2019 March 20, in College Station, Texas, USA. Further details about 2018 and 2019 G2F experiments can be found in Lima, Aviles, Alpers, McFarland, *et al*. (2023). The second population (Pop2) was developed by crossing doubled-haploid-derived inbred lines from the Wisconsin Stiff Stalk MAGIC population with PHZ51, a nonstiff stalk tester (Michel *et al*. 2022). Pop2 contains 220 hybrids that were planted on 2020 March 11 with drought (nonirrigated; 2020.drought) and optimal (2020.optimal) trials and on 2021 March 10 as drought (2021.drought) trial in College Station, Texas, USA. Further details about 2020 and 2021 G2F experiments can be found in Lima, Aviles, Alpers, Perkins, *et al*. (2023). Genomic data of the Pop1 and Pop2 were retrieved from the CyVerse platform (Lima, Washburn, *et al*. 2023). Following the exclusion of markers with a minor allele frequency below 5% and those with missing values exceeding 90%, 248,033 and 260,680 genomic markers were retained for Pop1 and Pop2.

### FHTP: drone, sensor, image processing, flight time, and data extraction

Red, green, and blue (RGB) field images were captured using a DJI Phantom 4 Pro V2.0, which was equipped with a 1-inch 20MP CMOS sensor featuring a mechanical shutter (manufactured by SZ DJI Technology Co., Ltd., Shenzhen, China). The flight missions, executed using the DJI GS Pro application, were planned at an elevation of 25 m above the ground with 90% forward and side overlaps, a flight speed of 1.2 m/s, and a shutter interval of 2.0 s. As a result, the individual images had a ground sampling distance of 0.7 cm/pixel.

Orthomosaics, a large image made out of the many smaller images captured by the drones’ camera, were necessary because each image had low spatial coordinate resolution and captured multiple plots per image. Orthomosaics were processed using Agisoft Metashape software (Agisoft LLC, St. Petersburg, Russia). Orthomosaics were created using the software's structure-from-motion technique combined with multiview stereo methodology. Ground control points were determined using a V-map Dual Frequency L1/L2 PPK GNSS Receiver from Micro Aerial Projects. The workflow for generating orthomosaics involved several steps: raw RGB images were loaded into an Agisoft project; the coordinate system and projection (commonly WGS84) were set; photos were aligned using referenced preselection, with key point and tie point limits set at 40,000 and 4,000, respectively; and an initial bundle adjustment was performed, optimizing camera calibration parameters such as focal length (*f*), optical center coordinates (*cx*/*cy*), and distortion coefficients (*k*1, *k*2, *k*3, *p*1, and *p*2). Tie point accuracy was improved by setting marker accuracy at 0.5 pixels and tie point accuracy at 0.1 pixels. Ground control points were imported as a .csv file and manually aligned. All images in the reference pane were unchecked, and camera alignment was optimized using all available distortion parameters. The bounding box was adjusted to encompass the entire sparse cloud; a dense cloud was generated. Colors were calibrated including white balance adjustments. A digital elevation map was built. Finally, orthomosaics were generated with the digital elevation map serving as the surface (https://www.agisoft.com/).

**Fig. 1.**
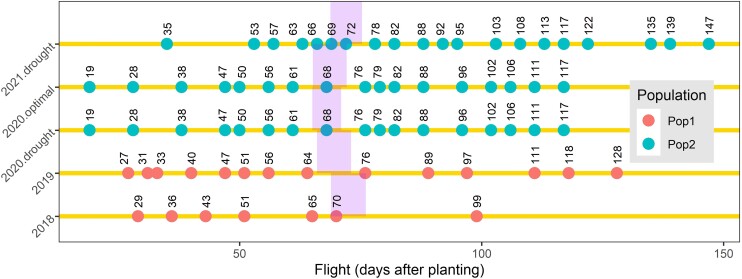
Flight times were illustrated as DAP unit for Pop1 and Pop2 grown in 2018–2019 and 2020–2021. The shading areas highlights the range of flowering times of plots in these trials.

The drone was flown at different dates during growth in each year and trial. Flight times were illustrated as days after planting (DAP) unit in Fig. 1 for Pop1 and Pop2 that were grown in 2018–2019 and 2020–2021, respectively. Overall, there were 7 and 14 flights for Pop1 grown in 2018 and 2019, respectively. There were 17 identical flights for Pop2 grown in 2020.drought and 2020.optimal trials due to the fact that each drone mission in 2020 contained both trials at each flight and 20 flights for the 2021.drought trial.

In total, 33 VIs along with R, G, and B bands (36 total, all will be referred to as VIs) were extracted from each orthomosaic (geotagged images). VIs were given in Supplementary Table 1. The polygons shapefiles (.shp) for the 2-row plots of each maize hybrid in each replication were generated using the R/UAStools package (Anderson and Murray 2020). These shapefiles were then utilized in R/FIELDimageR (Matias *et al*. 2020) to extract the VI values from each orthomosaics for each 2-row plot. The process involved using the R/FIELDimageR::fieldMask function to eliminate soil, based on color, from orthomosaics. Subsequently, all VIs were extracted from these orthomosaics for each flight date, using the R/FIELDImageR::fieldIndex function.

### Experimental design, phenotypes, and statistical assessment

Each trial was a randomized complete block design (RCBD) with 2 replications, employing a range-row grid planting. Each hybrid was planted as 2 consecutive row plots; each row plot had a length of approximately 8 m, and there was a gap of 0.8 m between the adjacent row plots.

Grain yield (t/ha) and plant height (cm) were collected from each row plot of Pop1 and Pop2 grown across 2018 and 2021. Grain yield was collected via a John Deere 3300 combined machine with a HarvestMaster grain gauge, while plant height data were collected at the end of growth manually and subjected to an ANOVA model (Equation 1) to estimate the genotypic effects (BLUEs) of hybrids in Pop1 and Pop2. This model was run for each population (Pop1 and Pop2) and year/trial (2018, 2019, 2020.drought, 2020.optimal, and 20201.drought) separately.


(1) Yijkl=μ+Hi+Rangej+Rowk+Repl+εijkl,

where $Y_{ijkl}$ is the GY or plant height of ith hybrid of Pop1 or Pop2 in the jth range, kth row, and lth replication. *μ* is the grand mean. *H* is the fixed effect of ith hybrid. Range and Row are lateral and horizontal grids constructing the RCBD design with *j* and *k* number of horizontal and lateral grids of regarding population and year, respectively, while Rep is the replication effect with *l* number of replications. $\varepsilon_{ijkl}$ is error. Equation (1) was also run with fully random effects to calculate the heritability for GY and plant height according to the following formula: $\mathrm{Heritability}\;=\frac{\sigma_{H_{i}}^{2}}{\sigma_{H_{i}}^{2}+(\sigma_{\varepsilon_{ijkl}}^{2}/2)}$ where $\sigma_{H_{i}}^{2}$ is the variance of hybrid effect and $\sigma_{\varepsilon_{ijkl}}^{2}$ is the variance of the error; the number of 2 represents the number of replications.

Values of the 33 VIs plus the R, G, and B bands belonging to each flight time (biomarkers) were subjected to the ANOVA model below (Equation 2) (Adak, Anderson, *et al*. 2023) to estimate the temporal value of each hybrid in Pop1 and Pop2 for each time point jointly in 2018–2021. The values of the biomarkers were analyzed using Equation (2) ANOVA model proposed by (Adak, Anderson, *et al*. 2023). This analysis aimed to estimate the temporal values of each hybrid in Pop1 and Pop2 across multiple time points of flights conducted from 2018 to 2021. Equation (2) model was run for each population (Pop1 and Pop2) and year/trial (2018, 2019, 2020.drought, 2020.optimal, and 2021.drought) separately. Equation (2) was run as a fully random effects model.


(2) Vijklm=μ+Hi+Fj+HFij+Rangek+Rowl+Repm+εijklm,

where $V_{ijklm}$ is the value of a VI belonging to ith hybrid of Pop1 or Pop2, jth flight (as DAP), kth range, lth row, and mth replication. $F_{j}$ is the random flight effect with *j* number of flights as DAP (Fig. 1). Rangek,Rowl,Repmandεijkl are same as those in Equation (1). *H* is the random effect of ith hybrid. As a result of $HF_{ij}$ component (temporal values as BLUPs) in Equation (2), Pop1 grown in 2018 produced 104,580 data points, derived from 415 hybrids, 36 VIs, and 7 flights. In 2019, Pop1 generated 209,160 data points with the same number of hybrids and VIs but doubled the flights to 14. Pop2, grown in drought trial in 2020, yielded 134,640 data points from 220 hybrids, 36 VIs, and 17 flights. Similarly, Pop2 grown in optimal trial in 2020 also resulted in 134,640 data points with the same number of hybrids, VIs, and flights. In 2021, Pop2, grown in the drought trial, resulted in 158,400 data points from 220 hybrids, 36 VIs, and 20 flights.

Temporal correlations were calculated between temporal values of each of 36 VIs with GY and plant height for each population in their growing environments. Means of temporal correlations were presented in this study.

### FPCA for temporal phenomic data

The temporal values ($HF_{ij}$; BLUPs) of each VI obtained by Equation (2) within every population and growth year/trial were analyzed using FPCA in the *fdapace* package (Zhou *et al*. 2022) in R based on the following formula:


(3) $$V_{i}(t)=\mu (t)+\overset{\infty }{\underset{i=1}{\sum }}\;\xi_{i}\varphi_{i}(t).$$

In this Equation (3), $V_{i}(t)$ represents the centered curve at flight time points, where *i* indices the temporal values of a VI, and *t* denotes the flight times. The term μ(t) refers to the mean curve, while $\xi_{i}$ represents the scores or coefficients linked to the *i*th functional principal component. Additionally, $\varphi_{i}(t)\;$ signifies the eigenfunctions corresponding to the *i*th functional principal component. The first 2 FPCA values of each VI were used in further analysis. Finally, FPCA1 and FPCA2 scores were calculated separately for each hybrid and VI in Pop1 and Pop2 in each year. In the FPCA, the temporal data of each VI data were tested in 2 ways separately, using only flights between planting and flowering and using all flights from planting to the end of the growth period. The period until flowering was chosen because the VIs visibly change at flowering and also because success in this early growth period would allow for earlier selection decisions and reduce the resources needed for flying. The ranges of flowering times were estimated by Equation (1) and were visually indicated in Fig. 1 with purple shading for each population and year/trial. For instance, in 2018, the FPCA involved examining the temporal data of each of the 36 VIs at specific DAPs: 29, 36, 43, 51, and 65. This period captured the flight times from the initial planting to the start of flowering (Fig. 1). The same FPC was also applied to the temporal data of the same set of VIs across all flights from 29 to 99 DAPs. Consequently, 2 distinct sets of phenomic data were created for Pop1 grown in 2018, each comprising 29,880 data points. One data set was generated using 415 hybrids, 36 VIs, and 2 FPCA scores calculated from flights occurring between planting and flowering initiation. The other data set utilized the same hybrids, VIs, and 2 FPCA scores but was calculated across all flights. Similar procedures were followed for Pop1 in 2019, Pop2 in both drought and optimal trials in 2020, and the drought trial in 2021; each was analyzed separately. Finally, as enabled by the calculation of the first (FPCA1) and second (FPCA2) functional principal component scores for temporal data in all trials, phenomic data of each of the 36 VIs were able to be merged row-wise owing to the presence of identical column names even though flight dates were not identical (Fig. 1). This enabled the calculation of a multiyear, multitrial phenomic relationship matrix as detailed in the following section.

### Prediction model

Six prediction models were developed, incorporating both single and multiple kernels utilizing phenomic and genomic data, either alone or in combination. Additionally, a multitrait prediction model was constructed. These models were employed to predict GY and plant height separately for Pop1 and Pop2. Prediction Model 1 (M1) focused on genomic prediction. Prediction Model 2 (M2) utilized phenomic prediction with FPCA1 and FPCA2 based on temporal data of all the VIs until flowering time. Prediction Model 3 (M3) also employed phenomic prediction with FPCA1 and FPCA2 but used temporal data of all VIs from planting until the end of the growth period. Prediction Model 4 (M4) was a multikernel prediction model incorporating both genomic and phenomic data used in M1 and M2. Prediction Model 5 (M5) was also a multikernel prediction model incorporating both genomic and phenomic data used in M1 and M3. Lastly, Prediction Model 6 (M6) involved multitrait genomic prediction, where the secondary trait was FPCA1 scores of visible atmospherically resistant index (VARI). The VARI was chosen for the example of a secondary phenomic trait because its temporal data provided the highest temporal correlations with both GY and plant height. FPCA1 of VARI was derived from using its temporal data belonging to flight times from planting to flowering to conduct the multitrait prediction until flowering time. Prediction models were given in Table 1:

**Table 1.** The prediction models with their notations.

Model name	Notation
M1: Genomic	$y_{i}=\mu +E+g+(g\times E)+\varepsilon$
M2: Early phenomic	$y_{i}=\mu +E+P_{1}+(P_{1}\times E)+\varepsilon$
M3: All phenomic	$y_{i}=\mu +E+P_{2}+(P_{2}\times E)+\varepsilon$
M4: Genomic + early phenomic	$y_{i}=\mu +E+g+P_{1}+(g\times E)+(P_{1}\times E)+\varepsilon$
M5: Genomic + all phenomic	$y_{i}=\mu +E+g+P_{2}+(g\times E)+(P_{2}\times E)+\varepsilon$
M6: Multitrait (phenotype + VARI FPCA1)	$y_{ij}=\mu +E+g+(g\times E)+\varepsilon$

*g* is the genomic relationship kernel; $P_{1}$ is the phenomic relationship kernel containing the flight information until flowering time, and $P_{2}$ is the phenomic relationship kernel containing the information of all flights in each environment. *E* is the environment matrix.

$y_{i}$ in M1–M5 is the vector of BLUEs values of GY or plant height of ith hybrid, while $y_{ij}$ in only M6 is the vector of multitrait vector of ith hybrid at jth traits. The BLUEs of either GY along with a secondary trait of a VI or plant height along with a secondary trait of a VI were used as the 2 trait combinations in multitrait prediction (M6). FPCA1 of the secondary VI trait was derived using the time points from planting to flowering time. *μ* is the overall mean of the given trait (GY or plant height). *E* is the effect of environment with $E∼\left(0,\frac{Z_{E}Z_{E}^{\prime }}{2}\right)$ for Pop1 and $E∼\left(0,\frac{Z_{E}Z_{E}^{\prime }}{3}\right)$ for Pop2 since Pop1 and Pop2 were grown in 2 years (2018 and 2019) and 3 environments (drought and optimal trials in 2020 and drought trial in 2021), respectively. $g=\;\{g_{j}\}\;∼\;N(0,G,\sigma_{g}^{2})$ is the genomic effect with $G∼\left(0,\frac{XX^{\prime }}{p}\right)$, where *p* is the number of markers of either Pop1 and Pop2, and ***X*** is the centered and standardized (by columns) matrix of genomic markers. *P* is the effect for phenomic data, and $P_{1}$ and $P_{2}$ were donated to phenomic effects that used flight times from the initial planting to the start of flowering or all flights, respectively. So $P∼\left(0,\frac{RR^{\prime }\sigma_{r}^{2}}{2\times 36}\right)$, where *R* is the relationship matrix for phenomic effect, 36 and 2 are the number of VIs (plus R, G, and B) and their 2 FPCAs scores, and $\sigma_{r}^{2}$ is the associated variance component. Interactions between *E* and *g* and *P* were defined according to Jarquín *et al*. (2014) and as follows: $g\times E\;∼\;\left(0,\frac{Z_{E}Z_{E}^{\prime }⊙Z_{g}GZ_{g}^{\prime }\sigma_{g\times E}^{2}}{\mathrm{no}\;\mathrm{of}\;\mathrm{envs}}\right)$ and $P\times E\;∼\;\left(0,\frac{Z_{E}Z_{E}^{\prime }⊙RR^{\prime }\sigma_{r\times E}^{2}}{no\;of\;envs\times 36\times 2}\right)$ with variance components of $\sigma_{g\times E}^{2}$ and $\sigma_{r\times E}^{2}$. Note that because these were top-crossed hybrids, it was not possible to estimate dominance effects in the model.

### Training models, prediction scenarios, and prediction ability

**Fig. 2.**
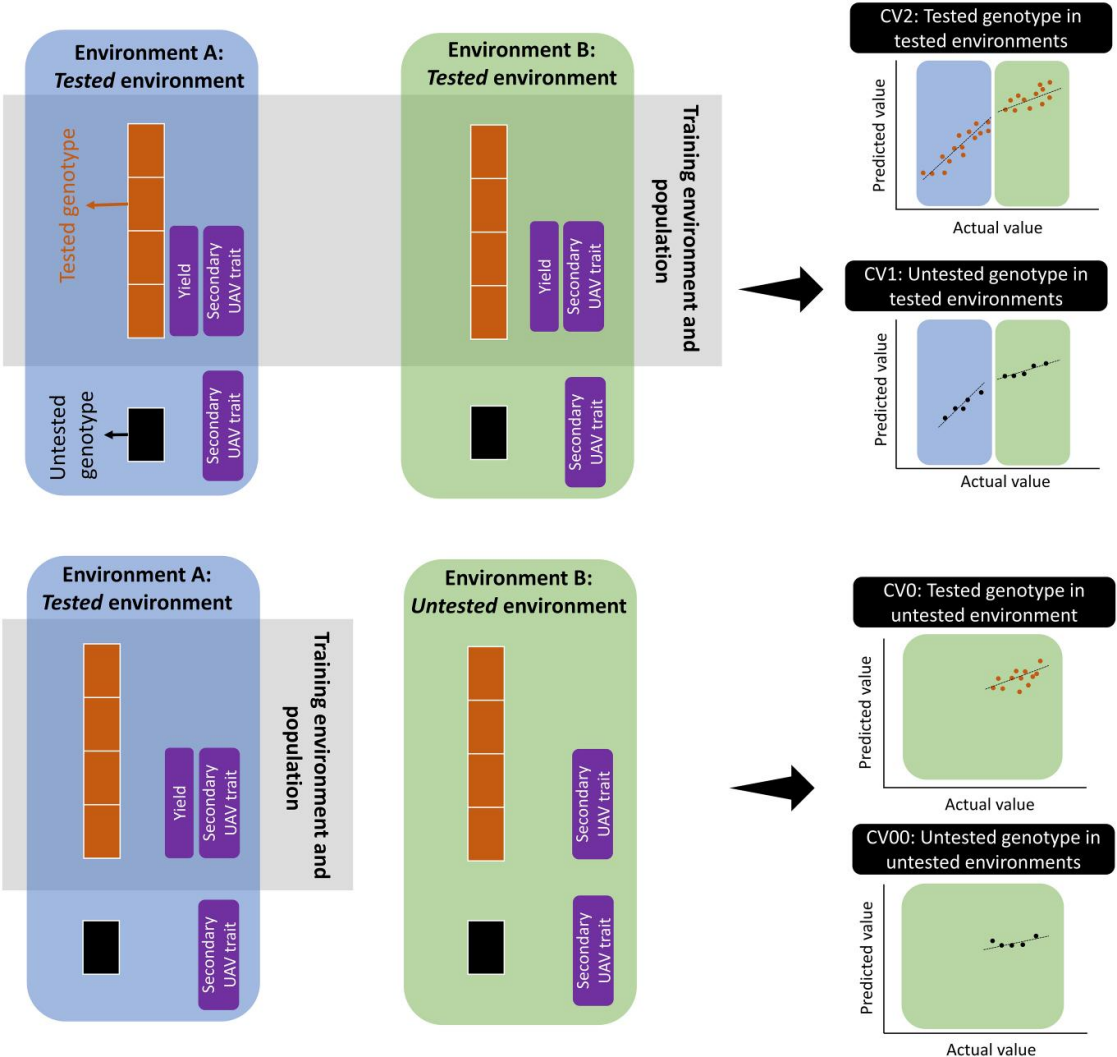
Model training scenarios, involving both tested and untested genotypes and environments, along with prediction scenarios. In the upper part of the figure, models were trained using a 4-fold genotype (represented by 4 orange blocks) in environments A and B. Subsequently, predictions were made for both tested and untested genotypes in both environments. The prediction scenarios CV2 and CV1 were then computed. In the lower part of the figure, the models were trained using the same 4-fold genotype, but exclusively in environment A (considered the tested environment). Following this training, predictions were generated for both tested and untested genotypes in environment B (considered the untested environment). The prediction scenarios CV0 and CV00 were then computed. A secondary trait (VARI FPCA1) was consistently present in the training set for M6 (multitrait prediction) across all prediction scenarios. Tested and untested hybrids were the same across all prediction scenarios (CV2, CV1, CV0, and CV00).

M1–M5 and M6 underwent training using the BGLR::BGLR() and BGLR::Multitrait() functions, respectively, within the BGLR package in R (Pérez and de los Campos 2014; Pérez-Rodríguez and de los Campos 2022). The training process involved employing a 5-fold cross-validation approach with 20 repeats. In each repeat, 4-folds were utilized for training the models and designated as the tested genotype (hybrid). Concurrently, the remaining 1-fold was excluded from the training data set and designated as the untested genotype. Thus, 332 and 176 hybrids in Pop1 and Pop2 corresponded to 4-fold tested hybrids respectively, while the remaining 83 and 44 corresponded to 1-fold untested hybrids. Four prediction scenarios relevant to plant breeding programs were applied to 6 prediction models, namely, CV1, CV2, CV0, and CV00 (Burgueño *et al*. 2012). In CV1 and CV2, all models were trained using the same tested genotypes within a population belonging to all environments. For instance, in Pop1, tested genotypes belonging to 2018 and 2019 were used to train the models. In Pop2, tested genotypes belonging to drought and optimal trials in 2020 and the drought trial in 2021 were used to train the models. So, correlations between predicted and actual values of the predicted trait were separately calculated for tested and untested genotype in each year/trial and denoted as CV2 and CV1, respectively (Fig. 2). In CV0 and CV00, all models were trained utilizing the tested genotype from training environments through the application of leave-one-out cross-validation for those environments. For example, the tested genotypes grown in the year 2018 were used to train the models in Pop1. Subsequently, predictions were made for both tested and untested genotypes in the year 2019, and the correlation between predicted and actual values for the predicted trait was separately computed for tested and untested genotypes, denoted as CV0 and CV00, respectively. In this context, 2018 and 2019 served as the tested and untested environments, respectively. The same hybrids were excluded from training and testing data sets to prevent genotype information leakage. Prediction was also carried out using 2019 as the tested environment and 2018 as the untested environment. Regarding Pop2, each potential pair of environments was utilized as the tested environment (for example, drought and optimal trials in 2020), with the remaining environment (for example, the drought trial in 2021) being assigned as the untested environment (Fig. 2). Predicted phenotypes used GY or plant height separately in M1–M5 models while each predicted phenotype was used along with the VARI VI FPCA1 scores in M6. Secondary traits were available for both test and untested genotypes and environments (Fig. 2).

## Results

### Grain yield and plant height variation and their heritabilities

**Fig. 3.**
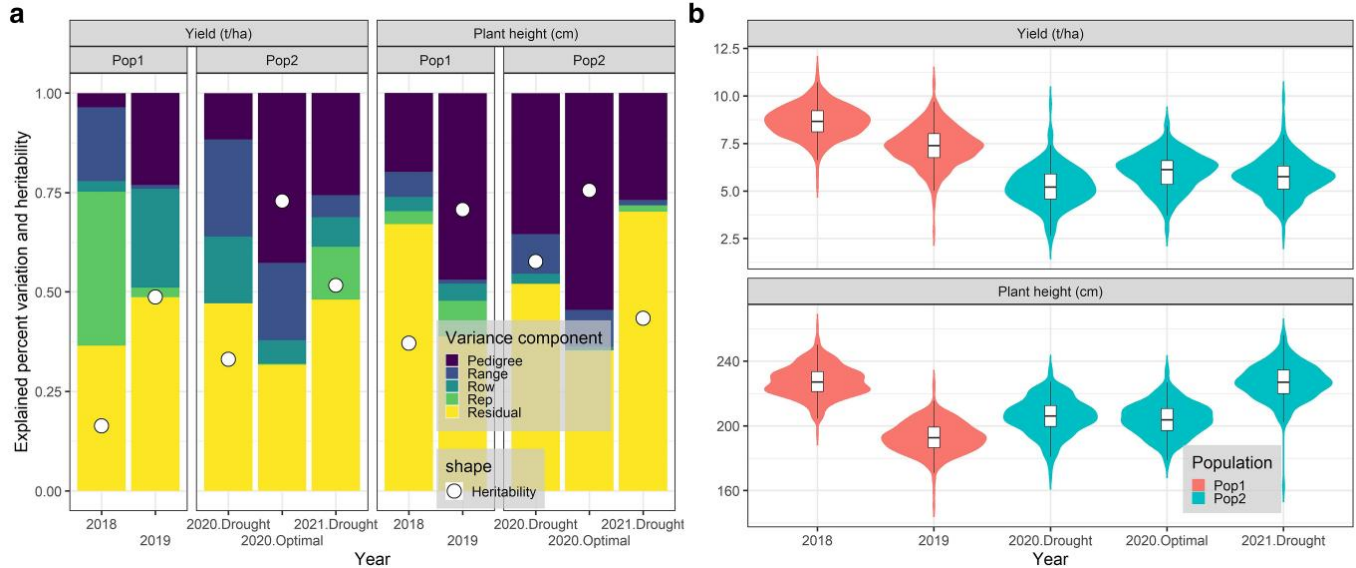
a) Explained percentage variation accounted for by the variance components in Equation (1), along with the heritabilities for plant height (in cm) and GY (in t/ha). b) Grain yield (t/ha) and plant height (cm) data range for Pop1 and Pop2 across 2018–2021. The *x* axis separates the growing trial and years for Pop1 and Pop2.

Grain yield and plant height data were collected at the conclusion of the growing season for Pop1 and Pop2 spanning the years 2018–2021, revealing heritabilities of GY between 0.16 and 0.73 and plant height between 0.37 and 0.76 depending on population and years (Fig. 3a). Variation in GY and plan height across different trials and years was observed (Fig. 3b). In Pop1, the GY was notably higher in 2018, reaching at 8.7 ± 0.8 t/ha, compared to the yield in 2019, which was 7.4 ± 1.0 t/ha. For Pop2, the optimal trial in 2020 exhibited the highest GY at 6.0 ± 1.0 t/ha, followed by 5.7 ± 1.0 t/ha and 5.2 ± 1.0 t/ha in drought trials in 2021 and 2020, respectively (Fig. 3b).

Regarding plant heights, Pop1 exhibited heights of 227.4 ± 9.6 cm in 2018 and 192.8 ± 9.4 cm in 2019. Pop2 displayed heights of 204.1 ± 10.2 cm, 205.8 ± 11.2 cm, and 227.1 ± 11.1 cm in optimal and drought trials in 2020 and drought trials in 2021, respectively (Fig. 3b).

### Temporal correlation between predicted variables and VIs

**Fig. 4.**
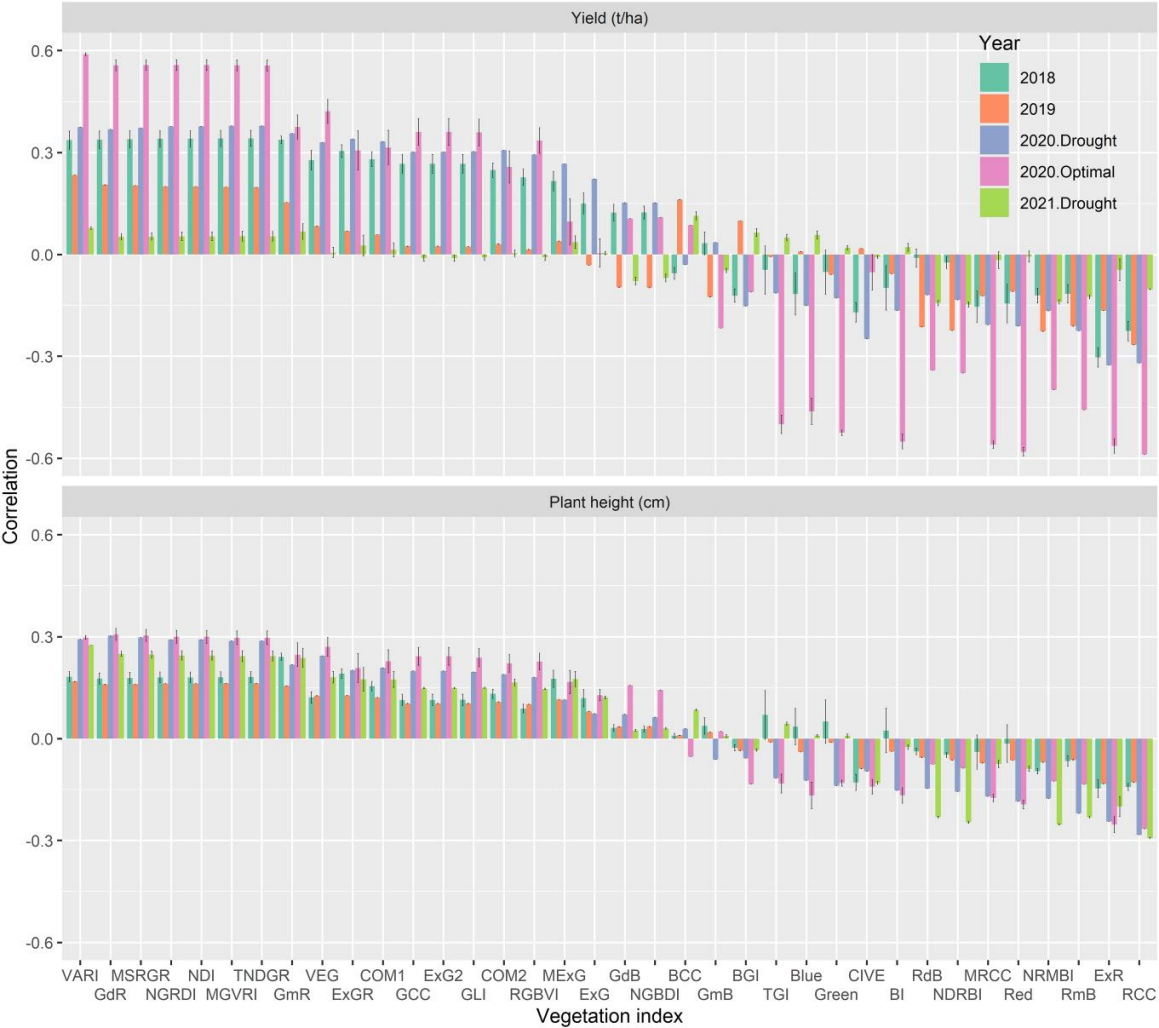
Temporal correlation results were illustrated between temporal values of each of 36 VIs and GY (below) and plant height (above) for each trial/year. Whiskers are the standard deviations of temporal correlations.

**Fig. 5.**
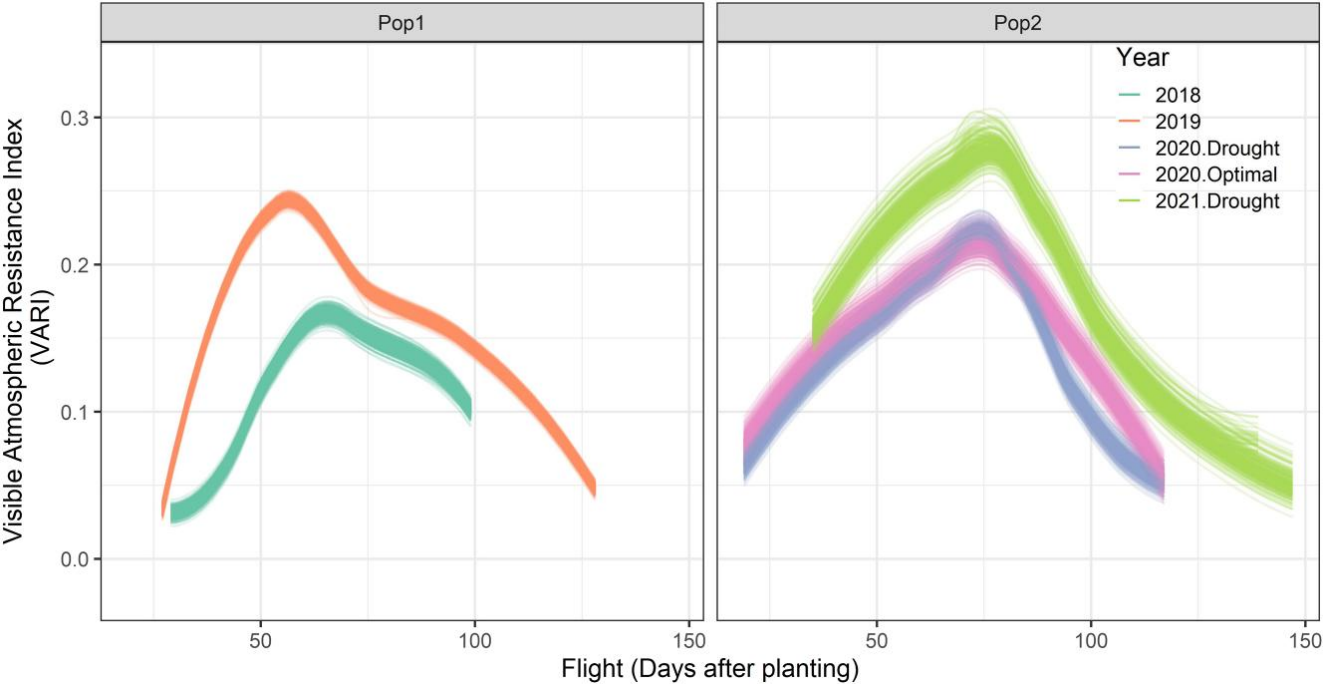
Temporal trajectories of VARI VI for Pop1 grown in 2018 and 2019 and Pop2 grown in drought and optimal trials in 2020 and drought trial in 2021. *y* axis is the value of the VARI; *x* axis is the days after planting.

Temporal correlations exhibited instability across different growing environments, as depicted in Fig. 4. Among VIs investigated, VARI always had strong temporal positive temporal correlations and red chromatic coordinate (RCC) index always had strong negative temporal correlations for both GY and plant height (Fig. 4). For instance, the mean of temporal correlations reached a peak at 0.59 ± 0.004, observed between VARI and GY in the optimal trial of 2020. Conversely, the most substantial negative temporal correlations were computed at −0.59 ± 0.000, involving the RCC index and GY in the optimal trial of 2020 (Fig. 4). The average temporal correlation with plant height was weaker, reaching approximately 0.3 and −0.3 with plant height for VARI and RCC, especially in Pop2 grown in drought and irrigated trials in 2020 and the drought trial in 2021, and further weakening in Pop1 grown in 2018 and 2019. As VARI showed the strongest positive temporal correlation with both GY and plant height, we examined temporal trends across flights. The analysis revealed distinct temporal trajectories for each growing environment (Fig. 5). In the M6 multitrait prediction, FPCA1 scores for VARI, calculated based on the time between planting and flowering, were selected as a secondary trait. This decision was influenced by the highest positive temporal correlation observed with both GY and plant height.

### Explained percent variation by functional principal competent for phenomic data

**Fig. 6.**
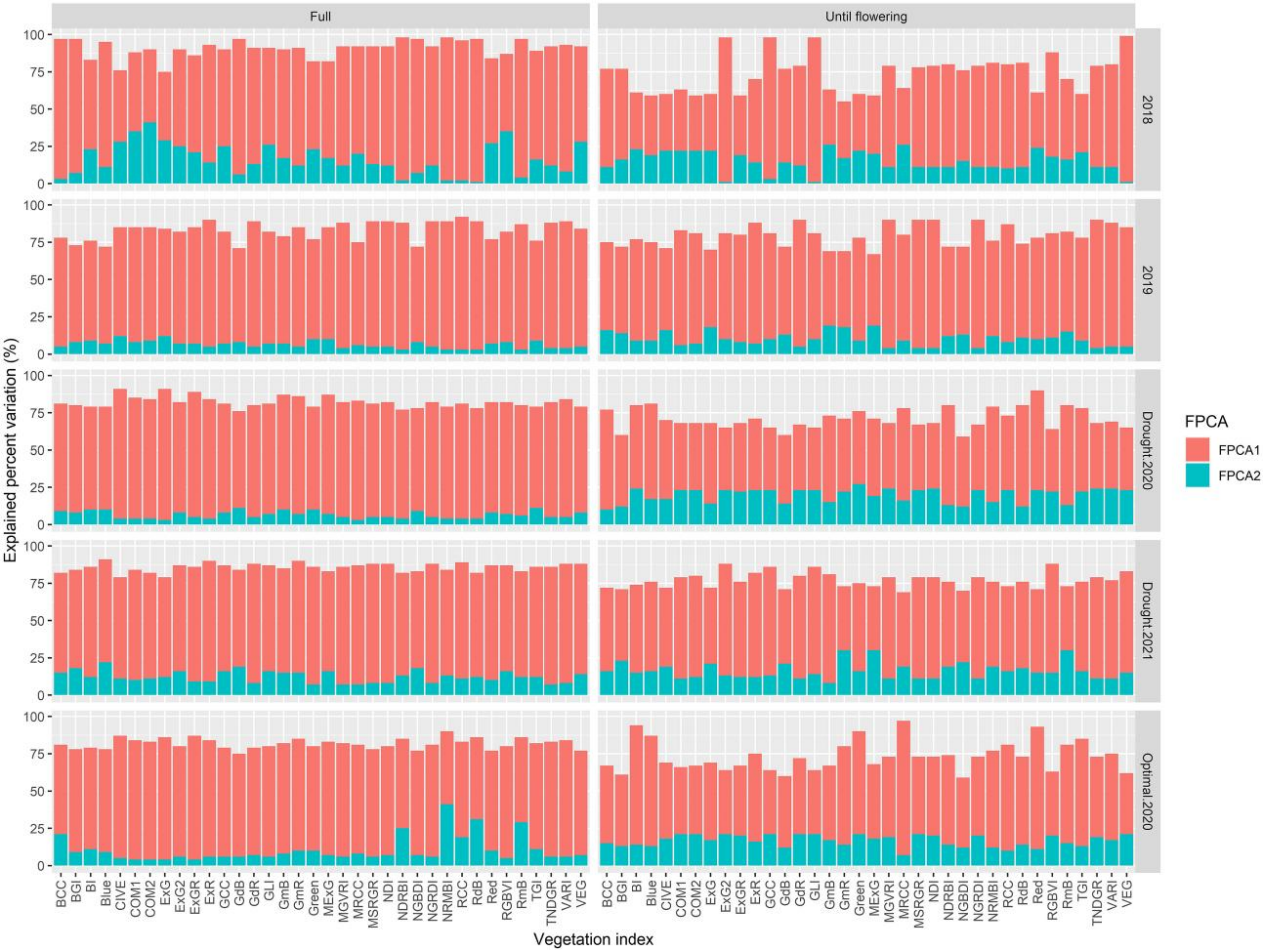
Explained percent variation by FPCA 1 and FPCA2 for temporal data of each of 36 VIs in each environment. On the left, FPCA1 and FPCA2 were obtained using temporal data of VIs belonging to all flights. On the right, FPCA1 and FPCA2 were obtained using temporal data of VIs belonging to planting to until flowering times.

The first 2 scores from FPCA were utilized for every VI to guarantee consistent column labels in the phenomic data throughout various growth sites. This standardization was crucial for creating subsequent phenomic relationship matrices, essential in predictive modeling. In general, these 2 FPCAs explained a substantial percentage of the variation across VIs. For example, when examining the temporal data of all VIs covering all flights and flights until flowering times, the explained percentage of variation by the first 2 FPCAs was 84.6% ± 5.6% and 74.8% ± 9.2% respectively (Fig. 6).

Furthermore, FPCA1 tended to account for the largest share of variation across all VIs when considering temporal data that included all flights until harvest, as opposed to data limited up until flowering (Fig. 6). For instance, FPCA1 explained 74.2%± 9.0% when utilizing all flights data, whereas it explained 59.3% ± 13.9% when using data only from planting until flowering. On the other hand, FPCA2 showed a higher degree of variation when considering temporal data until flowering, explaining 15.5% ± 6.1% compared to 10.4% ± 7.5% when using full data until harvest (Fig. 6).

### Phenomic, genomic, and combined prediction

**Fig. 7.**
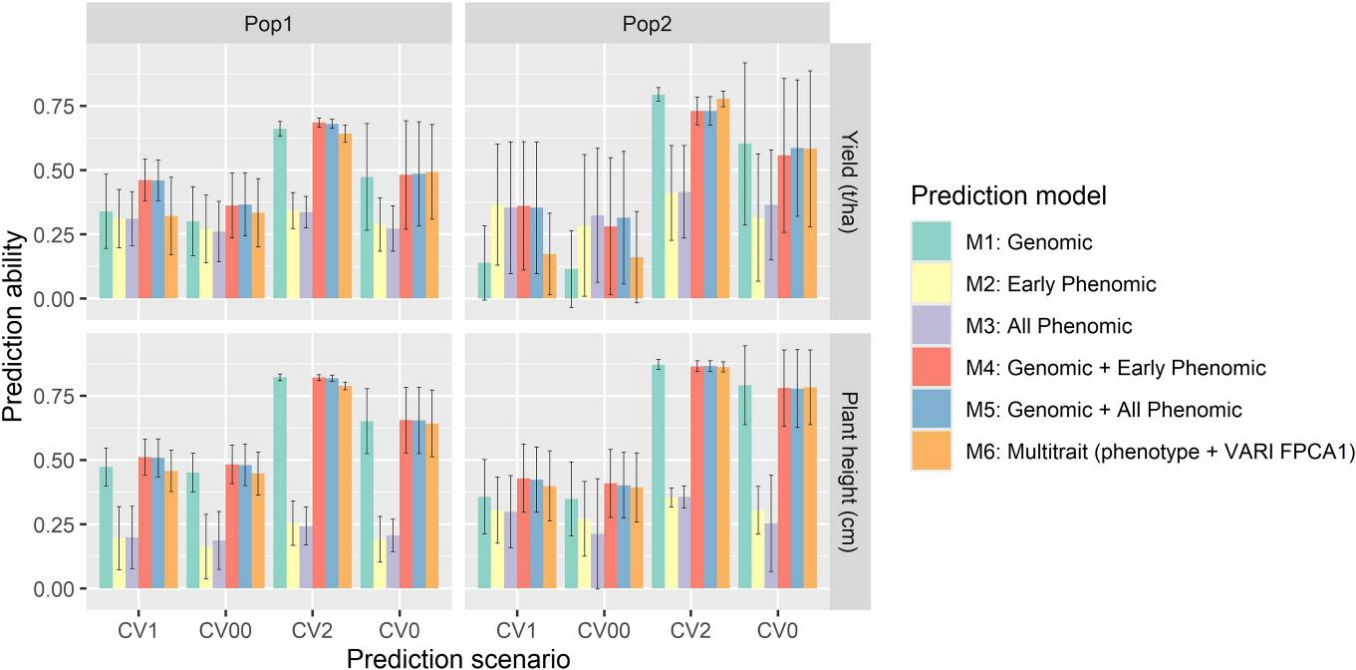
Prediction ability of the 6 prediction models for GY and plant height for Pop1 and Pop2. Bar plots represent the mean of prediction abilities along with the standard deviations (whiskers). M1 is the genomic prediction model. M2 and M3 are phenomic prediction models that use flight period data from planting to flowering and all flights, respectively. M4 and M5 are multikernel models that utilize M1 + M3 and M1 + M2 predictions, respectively. M6 is the multitrait (phenotype + VARI FPCA1) prediction model.

In predictive scenarios for plant breeding, particularly when predicting untested genotypes in both tested and untested environments (CV1 and CV00), the effectiveness of different models varies. For GY in Pop1, the genomic model (M1) and phenomic models (M2 and M3) showed similar performance. However, in Pop2, M2 and M3 slightly outperformed M1, although this advantage was not consistent across all testing scenarios. For plant height, M1 generally performed better than M2 and M3 in both populations. Nonetheless, the multikernel models (M4 and M5), which combine phenomic and genomic data, consistently showed the best overall performance for both GY and plant height. The multitrait genomic model (M6) performed comparably to M1 and was somewhat less effective than M4 and M5. It is important to note that differences in data density between genomic and phenomic predictions may impact these results, suggesting potential areas for future improvement. Despite these variations, the multikernel models demonstrate promising capabilities, particularly in some scenarios related to tested genotypes like CV2 and CV0, where they generally outperformed other models (refer to Table 2 and Fig. 7 for detailed results).

**Table 2.** Tabular format of prediction ability of the 6 prediction models for GY and plant height for Pop1 and Pop2.

Population	Model	CV2	CV1	CV0	CV00
Grain yield (t/ha)					
Pop1	M1	0.66 ± 0.03	0.34 ± 0.14	0.47 ± 0.21	0.30 ± 0.13
Pop1	M2	0.34 ± 0.07	0.31 ± 0.11	0.29 ± 0.10	0.27 ± 0.13
Pop1	M3	0.34 ± 0.06	0.31 ± 0.10	0.27 ± 0.09	0.26 ± 0.12
Pop1	M4	0.68 ± 0.02	0.46 ± 0.08	0.48 ± 0.21	0.36 ± 0.13
Pop1	M5	0.68 ± 0.02	0.46 ± 0.08	0.49 ± 0.20	0.37 ± 0.12
Pop1	M6	0.64 ± 0.03	0.32 ± 0.15	0.49 ± 0.18	0.33 ± 0.13
Pop2	M1	0.79 ± 0.03	0.14 ± 0.15	0.60 ± 0.32	0.11 ± 0.15
Pop2	M2	0.41 ± 0.18	0.36 ± 0.24	0.31 ± 0.25	0.28 ± 0.28
Pop2	M3	0.41 ± 0.18	0.35 ± 0.26	0.36 ± 0.21	0.32 ± 0.26
Pop2	M4	0.73 ± 0.05	0.36 ± 0.25	0.56 ± 0.30	0.28 ± 0.27
Pop2	M5	0.73 ± 0.06	0.35 ± 0.26	0.59 ± 0.27	0.31 ± 0.26
Pop2	M6	0.78 ± 0.03	0.17 ± 0.16	0.58 ± 0.30	0.16 ± 0.18
Plant height (cm)					
Pop1	M1	0.82 ± 0.01	0.47 ± 0.07	0.65 ± 0.13	0.45 ± 0.08
Pop1	M2	0.25 ± 0.09	0.20 ± 0.12	0.19 ± 0.09	0.16 ± 0.13
Pop1	M3	0.24 ± 0.07	0.20 ± 0.12	0.21 ± 0.06	0.19 ± 0.11
Pop1	M4	0.82 ± 0.01	0.51 ± 0.07	0.66 ± 0.13	0.48 ± 0.07
Pop1	M5	0.82 ± 0.01	0.51 ± 0.07	0.65 ± 0.13	0.48 ± 0.08
Pop1	M6	0.79 ± 0.02	0.46 ± 0.08	0.64 ± 0.13	0.45 ± 0.08
Pop2	M1	0.87 ± 0.02	0.36 ± 0.14	0.79 ± 0.15	0.35 ± 0.14
Pop2	M2	0.35 ± 0.04	0.30 ± 0.13	0.30 ± 0.09	0.27 ± 0.15
Pop2	M3	0.36 ± 0.04	0.30 ± 0.14	0.25 ± 0.19	0.21 ± 0.21
Pop2	M4	0.86 ± 0.02	0.43 ± 0.13	0.78 ± 0.15	0.41 ± 0.13
Pop2	M5	0.86 ± 0.02	0.42 ± 0.13	0.78 ± 0.15	0.40 ± 0.13
Pop2	M6	0.86 ± 0.02	0.40 ± 0.14	0.78 ± 0.15	0.39 ± 0.13

## Discussion

Comprehensive understanding and prediction of complex traits is a pivotal challenge in contemporary scientific research. The integration of phenomic data from field high-throughput phenotyping (FHTP) applications alone or integration with genomic data in multikernel-based prediction models offers promising avenues to enhance precision in predictive plant breeding methodologies and provide insights into their underlying biology. The convergence of multidimensional data not only underscores the advantage of leveraging new technologies but also highlights the potential synergistic effects arising from the fusion of diverse data sets. This synergy presents an advanced capacity to reliably predict and manipulate complex traits even in diverse and unobserved environments.

Phenomic data are a relatively recent addition in predictive breeding and genetics compared with genomic data, yet it necessitates further optimizations for its application within breeding programs. The phenomic traits extracted and used have so far been limited (VI's, plant height) to capture the phenome compared with the development of genetic markers to capture the genome. For example, here, there were 36 VIs by 7–20 flights depending on population and environment giving 252–720 phenomic feature biomarkers, compared with 248,033 and 260,680 DNA genomic markers for Pop1 and Pop2, around 3 orders of magnitude in difference. It is reasonable to expect the number of phenomic biomarkers will increase in the future, much as DNA genomic markers went from rare restriction fragment length polymorphisms 30 years ago to plentiful single nucleotide polymorphisms today. This increase could come spectrally (multispectral and hyperspectral), temporally (more flights throughout the growing season), and/or structurally through better resolution or algorithms for plant appearances, like texture (Wang, Yi, *et al*. 2021). A commensurate increase in phenomic markers to genomic levels would likely improve predictions substantially and make methods of applying phenomic markers across environments (as shown here) more important. To minimize the large *p* small *n* problem of more predictors than observations, DNA markers are now being preselected to increase reproducibility and minimize multicollinearity or based on additional information, such as non-synonymous mutations (Bian *et al*. 2014; Ramstein and Buckler 2022). It is conceivable that as more phenomic biomarkers are developed, a subset of the least correlated or most predictive across environments could be used to minimize overfitting.

In phenomic prediction of untested genotypes, plants must still be grown and flown to capture VIs, but no yield data need to be collected once a prediction model is trained. Currently, this is conceived as advantageous in breeding for 3 main reasons: timing of decisions, reducing costs of environments, and reducing error. In crop improvement, speed is important. When yield can be predicted from drone phenotypes in early growth stages, such as before flowering as tested here, prediction of the most favorable varieties can be made earlier. This can allow additional generations per year for off-season nurseries. In the case of Texas, we often have less than 2 weeks to harvest summer trials, analyze data, make decisions, and ship seed to a winter nursery. Toward reducing the costs of environments, the largest field expense is in maintaining and harvesting the crop, so more environments could be planted and remotely sensed (by drone or eventually satellite) to predict each genotype favorability across more environments, and then these experiments could be abandoned from further research investment. The reduced error comes from data on problematic plots, such as low populations (when soil is excluded, the inference is still made on whatever plants are present, although the response to density must then be considered), pest damage, or harvesting mistakes. These cases could supplement or correct existing combined harvest data. Furthermore, if an entire environment is lost to external factors before harvest, the environment could still be predicted. These applications could be applied to breeding programs at present, but biological discoveries are additionally being made by researchers using the same phenomic data. It is, therefore, important to demonstrate that phenomic prediction at this early methodological stage can compare with, complement, or possibly exceed the mature gold standard of genomic prediction, giving credence to the concept that there is novel value in collection of phenomic data.

The results here revealed varying levels of success among different models in predicting GY in maize based using different sets of variables and analytical approaches. Specifically, univariate phenomic prediction models (M2 and M3) utilizing the first 2 FPCA scores derived from temporal VIs numerically outperformed both univariate and multivariate genomic prediction methods in Pop2. However, in Pop1, the base genomic, phenomic, and multitrait prediction models exhibited close performance (Table 2 and Fig. 7).

These results suggest that employing multiple VIs might encompass diverse quantitative aspects of crop physiology across different management practices, environmental conditions, or years (Peng and Gitelson 2011). Nonetheless, accurately capturing plant biomass health before conducting further analyses remains crucial (Meyer and Neto 2008).

As a VI, VARI was linked to the ratio of green plant area, chlorophyll abundance, and crop stress in maize (Viña *et al*. 2004). Additionally, it was shown that VARI is the least susceptible to negative impacts from atmospheric effects (Gitelson *et al*. 2002). Other VIs like the normalized green red difference index have been associated with water use efficiency (Yang *et al*. 2020) and also are recognized as an important predictor of GY in maize (Chatterjee *et al*. 2023). This further reinforces the significance of diverse indices in predictive modeling. Moreover, RGB-derived VIs such as modified green red index and red green blue index were identified as early indicators of plant biomass (Bendig *et al*. 2015), underlining the importance of additional indices in capturing key growth stages. The triangular greenery index, proposed to link leaf chlorophyll content with close interactions of leaf nitrogen content (Hunt Jr. *et al*. 2011), adds to the array of VIs contributing to a comprehensive understanding of crop physiology.

Taken together, these diverse findings underscore the multifaceted nature of VIs in understanding and predicting GY as well as plant height in maize. This study uniquely provides a methodology to integrate temporal VIs across environments with disparate data collection. The collective body of research emphasizes the significance of an integrative approach, wherein the combination of VIs reflecting different physiological aspects such as chlorophyll abundance, plant biomass, water use efficiency, and nitrogen content can significantly enhance the accuracy and comprehensiveness of predictive models for maize yield estimation. These insights stress the necessity for a nuanced and comprehensive approach that accounts for the dynamic nature of crop physiology across diverse agricultural and environmental conditions, ultimately contributing to more reliable and robust phenotype predictions.

Multikernel prediction models (M4 and M5) with phenomic and genomic data showed the best potential for robust prediction in both GY and plant height of untested genotypes in either observed or unobserved and diverse environments (Table 2; Fig. 7). This result implies that the inclusion of both genomic and phenomic data in multikernel models can enable exploration beyond additive genetics such as epistasis and genetic–epigenetic interactions. Such interactions may play a critical role in how genetic factors manifest in observable traits in diverse environments, providing deeper insights into the complexity of the prediction of complex traits such as GY and plant height.

Previous studies have shown a benefit from using drone image features (e.g. VI) as a secondary trait in genomic prediction (Rutkoski *et al*. 2016; Sun *et al*. 2017; Sun, Poland, *et al*. 2019; Krause *et al*. 2020). In wheat, measuring secondary traits related to GY through a FHTP platform significantly boosted the accuracy of pedigree and genomic prediction models for GY on test sets; however, inconsistency in prediction accuracy across diverse environments was also reported (Rutkoski *et al*. 2016; Crain *et al*. 2018). Multivariate models were proposed as valuable predictions to perform selection in unobserved (untested) generations where accurately measuring GY is challenging, but secondary traits are observable by FHTP (Rutkoski *et al*. 2016; Sun *et al*. 2017). The FPCA approach presented here makes it possible to integrate data throughout the growing season.

Our multitrait genomic prediction model (M6) model agreed with previous studies since it numerically exceeded the base genomic prediction model (M1) in prediction the GY in Pop1 and Pop2 in the most challenging prediction scenario consistently (M1 vs M6 in CV00 in Table 2 and Fig. 7). In contrast to earlier research findings, our study utilized FPCA1 values obtained from the temporal data of the VARI belonging to vegetative growth stages. The VARI was chosen as a secondary trait in M6 since it showed the highest temporal correlation with GY (Fig. 4). This provided promising results since multitrait genomic prediction (M6) generally numerically performed comparably or outperformed the genomic model (M1) that was consistent across distinct populations (Pop1 and Pop2) under challenging predictive scenarios in different environments (CV00) (Table 2 and Fig. 7). Most VIs derived from various time points may capture the dynamic changes in plant growth throughout the growing season related to plant physiological processes such as environmental responses, stress tolerance, or overall plant health. As FPCA1 of the VARI captured temporal variation belonging to early flight dates before the onset of flowering times, it records physiological changes during vegetative growth stages. These FPCA1 scores could serve as early signals for both source-sink strength and the functional and visual aspects of “stay green” in maize, which are significant factors influencing eventual GY (Lee and Tollenaar 2007). FPCA scores will most substantially enhance the accuracy of predicting GY in new or unfamiliar environments when it is used as secondary trait in multitrait genomic prediction.

In conclusion, integrating multiple VIs can enhance predictive models’ robustness in estimating GY across diverse environmental conditions. The varying success of different prediction models across distinct populations highlights the importance of considering not only multiple VIs but also the timing of data collection of specific growth stages. Phenomic prediction models (M2) that used time points until flowering time offered earlier predictions of GY since M2 sometimes outperformed or performed similarly to M3. However, accurately capturing plant biomass and considering various VIs associated with different physiological aspects remain crucial for more accurate and comprehensive predictive modeling.

## Supplementary Material

jkae092_Supplementary_Data

## Data Availability

Supplementary files were stored in the “Data.zip” file containing the necessary files to reproduce the prediction codes. Data are available at figshare: https://doi.org/10.25387/g3.24657666. **FPCA.code.txt** contains the FPCA R code for a combination of growing environments (Pop1: 2018 and 2019 and Pop2: drought and optimal trials in 2020 and drought trial in 2021) and 2 different flight durations (flights until flowering time and full flights). **CSV files**: Files named FP.location.csv (e.g. FP.2018.csv) represent the results of flight–pedigree interaction in Equation (2) for each VI in each growing environment. Files named FP.location.FPCA.until_flowering.csv (e.g. FP.2018.FPCA.until_flowering.csv) contain the results of FPCA analysis using the flight information of each VI until flowering times in each growing environment. These files contain the first 2 FPCAs of each VI for each hybrid. Files named FP.location.FPCA.full_flights.csv (e.g. FP.2018.FPCA.full_flights.csv) present the results of FPCA analysis using all flight information of each VI in each growing environment. These files also contain the first 2 FPCAs of each VI for each hybrid. **GAPIT.Genotype.Numerical Pop1.txt and GAPIT.Genotype.Numerical Pop2.txt**: These files comprise the numeric genomic data of Populations 1 and 2, respectively. **PHT.csv and Yield.csv**: These files contain the plant height (cm) and grain yield (t/ha) BLUEs values for Pop1 and Pop2 grown in 2018 and 2019, drought and optimal trials in 2020, and the drought trial in 2021. They were utilized as predicted variables in the M1–M5 prediction models. **PHT.for.multitrait.csv and Yield.for.multitrait.csv**: These files contain the plant height (cm) and grain yield (t/ha) BLUEs values, along with the FPCA1 value of VARI as a secondary trait for Pop1 and Pop2 grown in 2018 and 2019 and drought and optimal trials in 2020, along with the drought trial in 2021. These data were used as predicted variables in the M6 prediction model. **Prediction code M1 to M5.txt and Prediction code M6.txt**: These files contain the prediction codes for M1–M5 and M6 prediction models, respectively. Supplemental material available at G3 online.
