# Supplementary material for: Field-based high-throughput phenotyping enhances phenomic and genomic predictions for grain yield and plant height across years in maize
Source: G3 (Bethesda). 2024 May 22;14(7):jkae092. doi: 10.1093/g3journal/jkae092 (PMC11228873; doi:10.1093/g3journal/jkae092)
Supplement: jkae092_Supplementary_Data [file jkae092_supplementary_data.docx]

**Supplementary table 1** shows the vegetation indices used and their formulas along with references.

| Vegetation index | Ratios | References |
| --- | --- | --- |
| **VIs derived from RGB bands** | | |
| Blue chromatic coordinate index (BCC) | $B/(R+G+B)$ | (Woebbecke et al., 1995) |
| Blue green pigment index (BGI) | $B/G$ | (Zarco-Tejada et al., 2005) |
| Brightness index (BI) | $sqrt((R^2+G^2+B^2)/3)$ | (Richardson and Wiegand, 1977) |
| Color index of vegetation extraction (CIVE) | $0.441R-0.811G+$  $0.385B+18.78745$ | (Kataoka et al., 2003) |
| Combined indices 1 (COM1) | $EXG+CIVE+EXGR+VEG$ | (Guijarro et al., 2011) |
| Combined indices 2 (COM2) | $0.36EXG+0.47CIVE+0.17VEG$ | (Guerrero et al., 2012) |
| Excessive green (ExG) | $2G-R-B$ | (Woebbecke et al., 1995) |
| Normalized Excess green index (ExG2) | $\frac{2G-R-B}{G+R+B}$ | (Woebbecke et al., 1995) |
| Excess green minus excess red index (ExGR) | $3G-2.4R-B$ | (Meyer and Neto, 2008) |
| Excessive red (ExR) | $1.4R-G$ | (Meyer et al., 1998) |
| Green minus blue index (GmB) | $G-B$ | (Woebbecke et al., 1995) |
| Green minus red index (GmR) | $G-R$ | (Woebbecke et al., 1995) |
| Green blue simple ratio index (GdB) | $\frac{G}{B}$ | (Woebbecke et al., 1995) |
| Green red simple ratio index (GdR) | $\frac{G}{R}$ | (Woebbecke et al., 1995) |
| Green chromatic coordinate index (GCC) | $G/(R+G+B)$ | (Woebbecke et al., 1995) |
| Green leaf index (GLI) | $(2G-R-B)/(2G+R+B)$ | (Louhaichi et al., 2001) |
| Modified excess green index (MExG) | $1.262G-0.884R$  $-0.311B$ | (Burgos-Artizzu et al., 2011) |
| Modified green red index (MGVRI) | $(G^2-R^2)/(G^2+R^2 )$ | (Bendig et al., 2015) |
| Modified Simple Ratio Green and Red (MSRGR) | $sqrt(G/R)$ | (Tucker, 1979) |
| Modified red chromatic coordinate index (MRCC) | $R^3/(R+G+B)$ | Created in this study |
| Normalized difference index (NDI) | $128*[(((G-R))/((G+R) ))+1]$ | (Meyer and Neto, 2008) |
| Normalized difference red  blue index (NDRBI) | $(R-B)/(R+B)$ | (Golzarian and Frick, 2011) |
| Normalized green-blue difference index (NGBDI) | $(G-B)/(G+B)$ | (Hunt et al., 2005) |
| Normalized green red difference index (NGRDI) | $(G-R)/(G+R)$ | (Tucker, 1979) |
| Normalized red minus blue index (NRMBI) | $(R-B)/G$ | Created in this study |
| Red minus blue index (RmB) | $R-B$ | (Woebbecke et al., 1995) |
| Red blue simple ratio index (RdB) | $R/B$ | (Woebbecke et al., 1995) |
| Red chromatic coordinate index (RCC) | $R/(R+G+B)$ | (Woebbecke et al., 1995) |
| Red green blue index (RGBVI) | $(G^2-R*B)/(G^2+R*B)$ | (Bendig et al., 2015) |
| Triangular greenery index (TGI) | $G-(0.39R-0.69B)$ | (Hunt et al., 2011) |
| Visible atmospherically resistant index (VARI) | $(G-R)/(G+R-B)$ | (Gitelson et al., 2002) |
| Transformed normalized green and red (TNDGR) | $sqrt(((G-R))/((G+R))+0.5)$ | (Tucker, 1979) |
| Vegetativen (VEG) | $G/(R^0.667*B^0.334 )$ | (Hague et al., 2006) |

R, G and B represent the red, green and blue reflectance bands respectively. Red, green and blue reflectance bands were also used in this study singly.

References

**Bendig J, Yu K, Aasen H, Bolten A, Bennertz S, Broscheit J, Gnyp ML, Bareth G** (2015) Combining UAV-based plant height from crop surface models, visible, and near infrared vegetation indices for biomass monitoring in barley. International Journal of Applied Earth Observation and Geoinformation **39:** 79-87

**Burgos-Artizzu XP, Ribeiro A, Guijarro M, Pajares G** (2011) Real-time image processing for crop/weed discrimination in maize fields. Computers and Electronics in Agriculture **75:** 337-346

**Gitelson AA, Kaufman YJ, Stark R, Rundquist D** (2002) Novel algorithms for remote estimation of vegetation fraction. Remote sensing of Environment **80:** 76-87

**Golzarian MR, Frick RA** (2011) Classification of images of wheat, ryegrass and brome grass species at early growth stages using principal component analysis. Plant Methods **7:** 1-11

**Guerrero JM, Pajares G, Montalvo M, Romeo J, Guijarro M** (2012) Support vector machines for crop/weeds identification in maize fields. Expert Systems with Applications **39:** 11149-11155

**Guijarro M, Pajares G, Riomoros I, Herrera P, Burgos-Artizzu X, Ribeiro A** (2011) Automatic segmentation of relevant textures in agricultural images. Computers and Electronics in Agriculture **75:** 75-83

**Hague T, Tillett N, Wheeler H** (2006) Automated crop and weed monitoring in widely spaced cereals. Precision Agriculture **7:** 21-32

**Hunt ER, Cavigelli M, Daughtry CS, Mcmurtrey JE, Walthall CL** (2005) Evaluation of digital photography from model aircraft for remote sensing of crop biomass and nitrogen status. Precision Agriculture **6:** 359-378

**Hunt ER, Daughtry C, Eitel JU, Long DS** (2011) Remote sensing leaf chlorophyll content using a visible band index.

**Kataoka T, Kaneko T, Okamoto H, Hata S** (2003) Crop growth estimation system using machine vision. *In* Proceedings 2003 IEEE/ASME International Conference on Advanced Intelligent Mechatronics (AIM 2003), Vol 2. IEEE, pp b1079-b1083 vol. 1072

**Louhaichi M, Borman MM, Johnson DE** (2001) Spatially located platform and aerial photography for documentation of grazing impacts on wheat. Geocarto International **16:** 65-70

**Meyer G, Hindman T, Laksmi K** (1998) MG (ed.), Deshazer JA, Machine vision detection parameters for plant species identification. Precision agri540 culture and biological quality, Boston, Massachusetts, USA **3:** 3543

**Meyer GE, Neto JC** (2008) Verification of color vegetation indices for automated crop imaging applications. Computers and electronics in agriculture **63:** 282-293

**Richardson AJ, Wiegand C** (1977) Distinguishing vegetation from soil background information. Photogrammetric engineering and remote sensing **43:** 1541-1552

**Tucker CJ** (1979) Red and photographic infrared linear combinations for monitoring vegetation. Remote sensing of Environment **8:** 127-150

**Woebbecke DM, Meyer GE, Von Bargen K, Mortensen DA** (1995) Color indices for weed identification under various soil, residue, and lighting conditions. Transactions of the ASAE **38:** 259-269

**Zarco-Tejada PJ, Berjón A, López-Lozano R, Miller JR, Martín P, Cachorro V, González M, De Frutos A** (2005) Assessing vineyard condition with hyperspectral indices: Leaf and canopy reflectance simulation in a row-structured discontinuous canopy. Remote Sensing of Environment **99:** 271-287
